# Complete genome sequence of *Rhodococcus ruber* SJ-1, a marine bacterium capable of degrading *p*-chloro-*m*-xylenol

**DOI:** 10.1128/mra.00675-25

**Published:** 2025-08-27

**Authors:** Jia Shi, Dan Xu, Qiao Ma

**Affiliations:** 1 Institute of Environmental Systems Biology, College of Environmental Science and Engineering, Dalian Maritime University 12421, Dalian, China; Montana State University, Bozeman, Montana, USA

**Keywords:** *Rhodococcus*, genome sequence, biodegradation, *p*-chloro-*m*-xylenol

## Abstract

We report the complete genome sequence of *Rhodococcus ruber* SJ-1, the first marine-derived bacterial strain capable of degrading the antibacterial agent *p*-chloro-*m*-xylenol. The genome consists of one chromosome (5,438,782 bp) and three plasmids (197,992 bp, 59,967 bp, and 41,343 bp), with an average GC content of 70.4%.

## ANNOUNCEMENT

**Fig 1:**
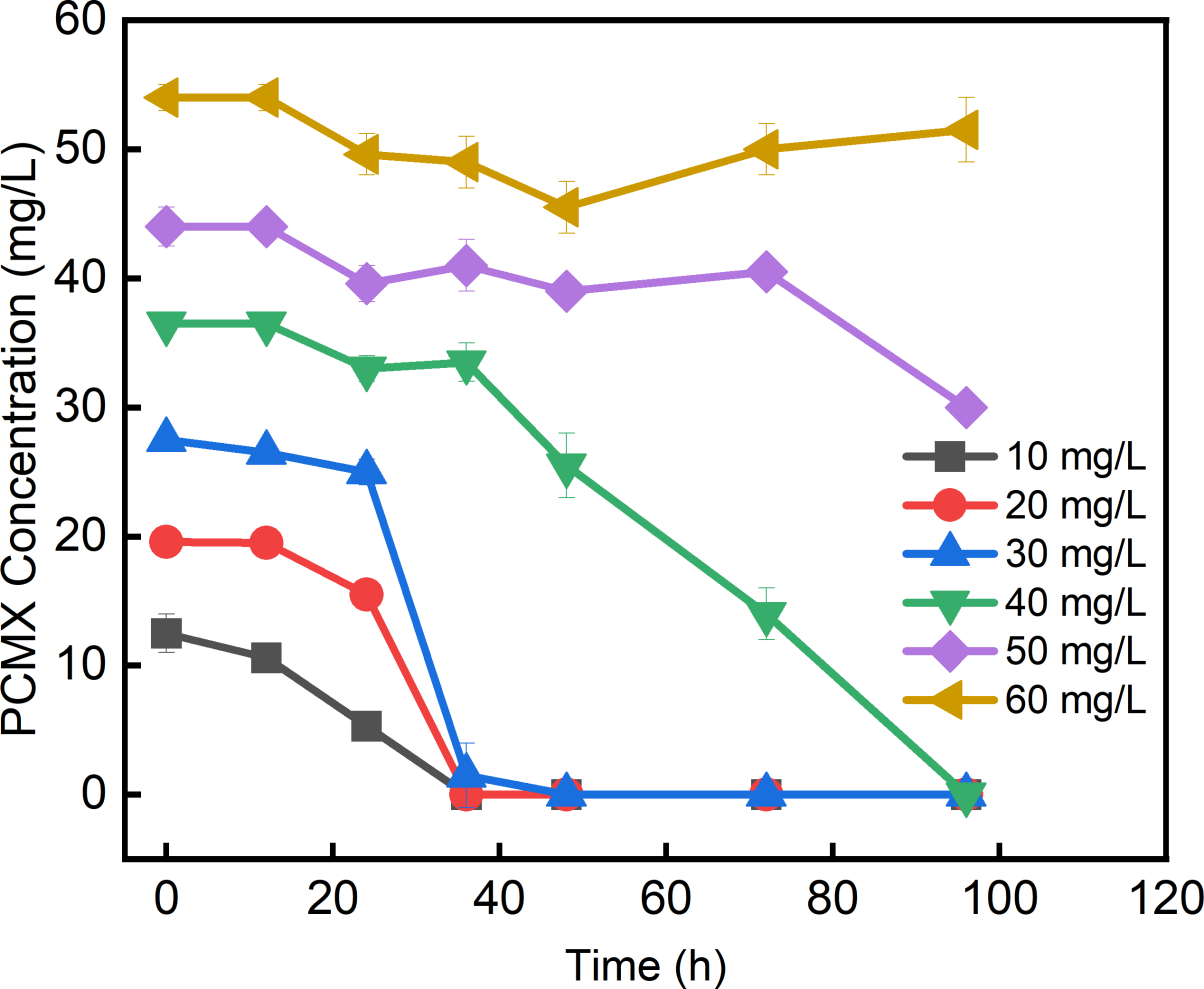
PCMX degradation curves by strain SJ-1 in 2216E medium

*Rhodococcus ruber* strain SJ-1 was isolated from a microbial consortium from sediment collected from the intertidal zone of Bohai Bay (E122.15^o^, N40.68^o^) (1). Strain isolation was performed using the conventional spread plate technique on the marine 2216E solid medium (30°C) supplemented with 30 mg/L *p*-chloro-*m*-xylenol (PCMX), a widely used antibacterial agent (2–4). The pure colony was transferred to 2216E liquid medium for degradation study. Strain SJ-1 demonstrated efficient PCMX degradation by removing 30 mg/L PCMX within 48 h under the conditions of 30°C and 150 rpm (Fig. 1). This study reports the first marine-derived *Rhodococcus* bacterium capable of degrading PCMX, which should provide valuable insights into the molecular mechanisms underlying its degradation (5–8).

Strain SJ-1 was cultured in 2216E medium until it reached the logarithmic growth phase. The bacterial cells were then harvested and washed with phosphate-buffered saline prior to DNA extraction. Genomic DNA was extracted using the FastPure Bacteria DNA Isolation Mini Kit (Vazyme, China), according to the manufacturer’s instructions. The integrity and purity of the DNA were assessed via 1% agarose gel electrophoresis. DNA concentration was quantified using Qubit 4.0 (Thermo Fisher Scientific, USA) and NanoDrop One (Thermo Fisher Scientific, USA). The genomic DNA was fragmented using a 26G needle, and fragments >20 kb were selected using the BluePippin system to prepare the SMRTbell DNA template library following the manufacturer’s instructions. Genomic sequencing was carried out following the standard protocol using the Pacific Biosciences Sequel IIe sequencer (PacBio, USA). Raw sequencing data were filtered, and genome assembly was performed using Canu v2.2 (9). Annotation and gene prediction from the assembled genome were performed using the NCBI Prokaryotic Genome Annotation Pipeline (10).

The raw data consisted of 56,172 reads, with an N50 read length of 6,389 bp. The complete genome comprised one circular chromosome and three plasmids, totaling 5,738,084 bp with 70.4% GC content (Table 1). Annotation predicted 5,256 protein-coding sequences (CDSs), 53 tRNA, and 12 rRNA genes. Phylogenetic analysis of the 16S rRNA gene sequences, performed using the molecular evolutionary genetics analysis software (MEGA, version 11), placed strain SJ-1 within the *R. ruber* clade among related strains retrieved from GenBank. Further validation by average nucleotide identity (ANI) analysis using the JSpeciesWS tool (https://jspecies.ribohost.com/jspeciesws/) confirmed this taxonomic assignment, revealing 99.2% similarity between SJ-1 and the type strain *R. ruber* NBRC 15591 (11, 12). This value substantially exceeded the established 95% ANI threshold for species delineation. Collectively, these genomic and phylogenetic data identified strain SJ-1 as *R. ruber*. Genomic annotation identified 19 putative CYP450 genes in strain SJ-1. Additionally, five genes encoding catechol *meta*-cleaving dioxygenase and catechol *ortho*-cleaving dioxygenase were detected. These genes and their associated clusters were likely involved in PCMX metabolism (5, 13), and functional validation studies were ongoing. Our findings establish critical genomic foundations for elucidating molecular mechanisms of PCMX catabolism in marine bacteria.

**TABLE 1:** General features of *Rhodococcus* sp. SJ-1

Item	Description
Cell shape	Rod-shaped
Colony color	Orange red
BioProject ID	PRJNA1171201
BioSample	SAMN44238121
GenBank accession number	CP194413-CP194416
SRA accession number	SRR34347629
Marine Culture Collection of China (MCCC)	MCCC 1K09480
Chromosome	5,438,782 bp
Plasmid 1	197,992 bp
Plasmid 2	59,967 bp
Plasmid 3	41,343 bp
Predicted CDSs	5,256
GC content (%)	70.4
tRNA genes	53
rRNA genes	12

## Data Availability

The complete genome sequence has been deposited in GenBank under the accession numbers CP194414.1 (chromosome), CP194413.1 (plasmid 1), CP194415.1 (plasmid 2), and CP194416.1 (plasmid 3). The BioProject accession number is PRJNA1171201, and the BioSample accession number is SAMN44238121. The raw sequencing reads have been deposited in the NCBI Sequence Read Archive (SRA) under the accession number SRR34347629.
